# Effect of 3-Month Aerobic Dance on Hippocampal Volume and Cognition in Elderly People With Amnestic Mild Cognitive Impairment: A Randomized Controlled Trial

**DOI:** 10.3389/fnagi.2022.771413

**Published:** 2022-03-10

**Authors:** Yi Zhu, Yaxin Gao, Chuan Guo, Ming Qi, Ming Xiao, Han Wu, Jinhui Ma, Qian Zhong, Hongyuan Ding, Qiumin Zhou, Nawab Ali, Li Zhou, Qin Zhang, Ting Wu, Wei Wang, Cuiyun Sun, Lehana Thabane, Ling Zhang, Tong Wang

**Affiliations:** ^1^Rehabilitation Medicine Center, The First Affiliated Hospital of Nanjing Medical University, Nanjing, China; ^2^Rehabilitation Medicine Center, The Affiliated Suzhou Hospital of Nanjing Medical University, Suzhou Municipal Hospital, Gusu School, Nanjing Medical University, Suzhou, China; ^3^Department of Radiology, The First Affiliated Hospital of Nanjing Medical University, Nanjing, China; ^4^Jiangsu Key Laboratory of Neurodegeneration, Center for Global Health, Nanjing Medical University, Nanjing, China; ^5^Brain Institute, the Affiliated Nanjing Brain Hospital of Nanjing Medical University, Nanjing, China; ^6^Rehabilitation Department, Nanjing Drum Tower Hospital, The Affiliated Hospital of Nanjing University Medical School, Nanjing, China; ^7^Department of Health Research Methods, Evidence, and Impact, McMaster University, Hamilton, ON, Canada; ^8^First School of Clinical Medicine, Nanjing Medical University, Nanjing, China; ^9^Swat Institute of Rehabilitation and Medical Sciences, Swat, Pakistan; ^10^Department of Neurology, The First Affiliated Hospital of Nanjing Medical University, Nanjing, China; ^11^Biostatistics Unit, St Joseph’s Healthcare, Hamilton, ON, Canada

**Keywords:** cognitive impairment, dancing, hippocampus, cognition, motor cognitive training

## Abstract

As an intermediate state between normal aging and dementia, mild cognitive impairment (MCI), especially amnestic MCI (aMCI), is a key stage in the prevention and intervention of Alzheimer’s disease (AD). Whether dancing could increase the hippocampal volume of seniors with aMCI remains debatable. The aim of this study was to investigate the influence of aerobic dance on hippocampal volume and cognition after 3 months of aerobic dance in older adults with aMCI. In this randomized controlled trial, 68 elderly people with aMCI were randomized to either the aerobic dance group or the control group using a 1:1 allocation ratio. Ultimately, 62 of 68 participants completed this study, and the MRI data of 54 participants were included. A specially designed aerobic dance routine was performed by the dance group three times per week for 3 months, and all participants received monthly healthcare education after inclusion. MRI with a 3.0T MRI scanner and cognitive assessments were performed before and after intervention. High-resolution three-dimensional (3D) T1-weighted anatomical images were acquired for the analysis of hippocampal volume. A total of 35 participants (mean age: 71.51 ± 6.62 years) were randomized into the aerobic dance group and 33 participants (mean age: 69.82 ± 7.74 years) into the control group. A multiple linear regression model was used to detect the association between intervention and the difference of hippocampal volumes as well as the change of cognitive scores at baseline and after 3 months. The intervention group showed greater right hippocampal volume (β [95% CI]: 0.379 [0.117, 0.488], *p* = 0.002) and total hippocampal volume (β [95% CI]: 0.344 [0.082, 0.446], *p* = 0.005) compared to the control group. No significant association of age or gender was found with unilateral or global hippocampal volume. There was a correlation between episodic memory and intervention, as the intervention group showed a higher Wechsler Memory Scale-Revised Logical Memory (WMS-RLM) score (β [95% CI]: 0.326 [1.005, 6.773], *p* = 0.009). Furthermore, an increase in age may cause a decrease in the Mini-Mental State Examination (MMSE) score (β [95% CI]: −0.366 [−0.151, −0.034], *p* = 0.002). In conclusion, 3 months of aerobic dance could increase the right and total hippocampal volumes and improve episodic memory in elderly persons with aMCI.

**Clinical Trial Registration:** This study was registered on the Chinese Clinical Trial Registry [www.chictr.org.cn], identifier [ChiCTR-INR-15007420].

## Introduction

Mild cognitive impairment (MCI) refers to the transitional stage between normal aging and probable Alzheimer’s disease (AD). In this stage, a person has impaired cognitive function unlike normal age-related cognitive decline, but it is not severe enough to cause significant impairment in the activities of daily life (Petersen et al., 1997). Those with amnestic MCI (aMCI), a subtype of MCI, are 4–10 times more likely to progress to AD than healthy elderly people (Petersen et al., 2001; Bischkopf et al., 2002; Grundman et al., 2004). The early diagnosis of and intervention in aMCI are key to prevent AD.

So far, there is no strong evidence of pharmaceutical treatment reversing the progression of AD (Wang et al., 2016; Fink et al., 2018). However, the role of non-pharmaceutical therapy in MCI prevention and treatment has been recognized, which includes change in lifestyle, the Mediterranean diet (Radd-Vagenas et al., 2018), risk factor control (Ganguli et al., 2013; Sanford, 2017), cognitive training (Hampstead et al., 2012), psychological intervention, and exercise therapy (Verdelho et al., 2013; Hsu et al., 2018; Zhu et al., 2020). Furthermore, motor cognitive training, which incorporates physical and cognitive tasks, is reported to be a more promising approach compared to independent physical or cognitive tasks (Herold et al., 2018). Dancing is a special type of motor cognitive training that combines physical activity with motor learning, attention, music, and rhythm–motor integration (Rektorova et al., 2020). Our recent meta-analysis concluded that dancing could improve cognition in elderly people with MCI (Zhu et al., 2020). Dance intervention has been reported to lead to intervention-specific complex brain plasticity related to cognitive performance (Balazova et al., 2021), so it is a promising candidate for counteracting the age-related decline in physical and mental abilities (Rehfeld et al., 2017). Meanwhile, hippocampal atrophy/shape change is believed to be a typical MRI marker in AD and aMCI (Shi et al., 2009; Frisoni et al., 2010), and hippocampal volume is a strong predictor of memory decline in MCI (Mak et al., 2017). In addition, a pair of studies showed that aerobic exercise could increase hippocampal volume in younger adults (Frodl et al., 2020) and people with MCI (ten Brinke et al., 2015), while another study revealed that dance-related cognitive improvement was not dependent on hippocampal atrophy in mixed seniors with normal cognition and MCI (Kropacova et al., 2019). Whether dancing could increase the hippocampal volume in patients with aMCI remains debatable, so we aimed to explore the change in hippocampal volume in patients with aMCI after aerobic dance intervention with a single-blinded, randomized controlled trial.

## Methods

### Study Design

This study is a single-blinded, randomized controlled trial to investigate the effects of aerobic dance and a health education program in older adults with aMCI. The Ethics Committee of the First Affiliated Hospital of Nanjing Medical University approved this study in January 2013 (2012-SR-098). All participants signed written informed consent forms.

### Sample Size Calculation

Changes in hippocampal volume on the right side after 3 months of intervention were considered the primary outcome for sample size calculation. To detect a moderate effect size of 0.75 standard deviation (SD), a minimum sample size of 56 (28 per group) was required to achieve 80% statistical power at the significance level of 0.05 (two-sided). The sample size was calculated using PASS version 16. Considering 20% potential loss to follow-up, a total of 68 participants were recruited for this trial.

### Patients

All participants were recruited from the memory clinic of the First Affiliated Hospital of Nanjing Medical University in the period from June 2014 to December 2016. The trial was approved by the Ethics Committee of the First Affiliated Hospital of Nanjing Medical University (Jiangsu Province Hospital, China). The neurologist of the memory clinic screened the patients with memory complaints. If a patient met the inclusion criteria, he/she was recruited into this study.

*Inclusion criteria* include those who (1) aged between 50 and 85 years (both inclusive); (2) met the diagnostic criteria of aMCI according to the National Institute of Aging and Alzheimer’s Association (NIA-AA) guidelines (Jack et al., 2018); (3) had experienced memory loss for at least 3 months; (4) had Mini-Mental State Examination (MMSE) score ≥ 25 and Montreal Cognitive Assessment (MoCA) score ≤ 26; (5) had Hachinski Ischemic Score (HIS) ≤ 4; (6) had above primary school education; and (7) provided written informed consent.

*Exclusion criteria* include those who (1) were diagnosed with vascular dementia based on the National Institute of Neurological Disorders and Stroke and the Association Internationale pour la Recherché et l’Enseignement; (2) had HIS > 4; (3) were unable to take the cognitive assessments and MRI tests due to disorders, such as deafness, blindness, or severe language disorders; (4) had drug intake in the past 6 months, which could influence cognitive performance; (5) had psychiatric problems, including severe depression or anxiety; (6) had medical contraindication of exercise, such as unstable conditions (e.g., cerebrovascular disease, liver and kidney disease, falling sickness, or disease of internal secretion); (7) had functional limitations caused by orthopedic diseases (e.g., fracture, osteoarthritis, or joint replacement); and (8) had long-term habit of dancing.

### Randomization

Participants were randomized in a 1:1 ratio and assigned to either the intervention group (e.g., aerobic dance and health education) or the control group (e.g., health education program only) based on a computer-generated randomized sequence by an independent statistician. Thereafter, a clinician who was not involved in the enrollment or outcome measures opened the sequentially numbered, sealed envelopes that had the details of the participants and their allocation to the respective groups.

### Interventions

#### The Intervention Group (Aerobic Dance Group)

The intervention group participated in a moderate-intensity group aerobic dance program for 3 months. The dance routine,^1^ which was designed by an experienced physical therapist (PT), lasted for approximately 35 min, and was performed three times per week. The dance routine included a 5-min warm-up, a 25-min dance with a target heart rate, and a 5-min cool-down period. The intensity of dancing was set to 60–80% of the maximum heart rate to ensure the safety of participants. Two PTs with more than 5 years of experience in exercise intervention administered the dance routine. One PT led the group dance, and the other one was responsible for monitoring heart rate and dancing performance. During the training, cardiotachometers (ONrhythm 50, GEONATURE) were used to monitor the heart rate of each participant. Each training session consisted of 11–16 participants, and during the first 2 weeks, the PT demonstrated a sequence of dancing steps and taught the participants how to combine the steps and follow the music. This dance routine was composed of seven subsessions, namely, knee bending, heel up, boxing, shoulder movement, kicking, square-stepping, and sculling exercises. All the participants had to highly focus during the training, memorize all the steps involved, and follow the movement sequence properly.

Both the intervention and control groups received a health education program (in the form of a 120-min-long lecture) after inclusion in this study. This education program covered information about risk factors of dementia, healthy diet, healthy lifestyle, and insomnia management. The participants were contacted by phone every week to remind them of the main points of the education program.

#### The Control Group

The control group received health education only.

### Outcome Measurements

All the participants were assessed for the primary and secondary outcome measurements at baseline and after 3 months of intervention. The primary outcome measure was the unilateral hippocampal volume, and the secondary outcomes were clinical assessments.

### MRI Acquisition and Analysis

The MRI scanning was performed on a 3.0T MRI System (Siemens AG, Erlangen, Germany) using a standard birdcage head transmit and receive coil at baseline and after 3 months of intervention. High-resolution three-dimensional (3D) T1-weighted anatomical images were acquired in the sagittal plan using a magnetization-prepared rapid gradient-echo sequence [repetition time (TR) = 1,900 ms; echo time (TE) = 2.52 ms; flip angle (FA) = 90°; field of view (FOV) = 256 mm × 256 mm; matrix size = 256 × 256; slice thickness = 1 mm; inter-slice gap = 0.5 mm; voxel size = 1 mm^3^; 176 slices]. After that, axial fluid-attenuated inversion recovery images were obtained for diagnosis, namely, inversion time (TI) = 2,500 ms; TR = 9,000 ms; TE = 100 ms; and slice thickness = 5 mm.

All MRI images were collected by a single imaging technologist and evaluated by an experienced radiologist to exclude patients with obvious brain lesions, such as cerebral infarction, moderate to severe white matter lesions assessed by the Fazekas scale (grades from 0 to 6), brain tumor, and other brain damage.

### Hippocampal Volume Calculation

T1-weighted structural images were analyzed for the measurement of hippocampal volume. The key to measuring hippocampal volume is to accurately demarcate by taking an oblique coronal section perpendicular to the long axis of the hippocampus and from the head to the tail of the hippocampus as the main measurement section (Cendes et al., 1993; Pruessner, 2000; Pruessner et al., 2001). We used two methods to measure hippocampal volume. The first method was manual measurement. Manual segmentations of unilateral hippocampal volume were obtained using the European Alzheimer’s Disease Consortium (EADC) – Alzheimer’s Disease Neuroimaging Initiative (ADNI) Harmonized Protocol (HarP; Frisoni et al., 2015). The measurements mainly followed the harp manual, and the human brain atlas was referred to if necessary determine the correct anatomical recognition. First, we identified the boundaries of the hippocampus through the sagittal and coronal plane and drew an outline of the structure of the hippocampus. Second, we calculated the area of each layer, multiplied the area of the hippocampus by the thickness of each layer, and summed the results to get the whole volume of the hippocampus. Then, we calculated the intracranial volume (ICV) and obtained standardization through the division of the hippocampal volume by the total brain volume (Free et al., 1995). The second method used the ITK-SNAP program to calculate the volume, which provided semi-automatic segmentation and manual delineation to analyze medical images and obtain their 3D models. We identified and labeled the borders of the left and right hippocampus separately for each image, and then we calculated the volume using some ITK-SNAP software tools (Yushkevich and Gerig, 2017).

One experienced investigator, who was blinded to the groups, measured the hippocampal volumes of both sides at baseline and after 3 months of dance intervention of all participants in random order using the two methods described.

### Clinical Assessments

Global cognition was assessed using MMSE and MoCA (Luis et al., 2009; Yu et al., 2012). Cognitive domains were assessed by various tests, including episodic memory [Wechsler Memory Scale-Revised Logical Memory (WMS-RLM); Wang et al., 2015], executive function [Trail Making Test Part A&B (TMT A&B); Perrochon and Kemoun, 2014], the Symbol Digit Modalities Test (SDMT; Cherbuin et al., 2010), and the Forward and Backward Digit Span Task (DST) Chinese version (Laures-Gore et al., 2011). Abilities of daily living were measured by the Functional Activities Questionnaire (FAQ), quality of life was measured by the 36-Item Short-Form Health Survey (SF-36; Lam et al., 1998; Hsiao et al., 2015), and depression was assessed using the Geriatric Depression Scale (GDS-15; de Paula et al., 2016). All these assessments were completed at baseline and after 3 months of dance intervention. The cognitive assessments were completed by an experienced speech therapist, and all other measurements were taken by an experienced physiatrist. Both of them were blinded to the randomization.

### Statistical Analysis

All data were analyzed using SPSS software version 25.0 (SPSS Inc., Chicago, IL, United States). The distribution of all variables was inspected using descriptive statistics. An independent sample *t*-test was applied to assess differences in age and weight between the intervention group and the control group. A chi-square test was used to assess differences in the distribution of HIS and the prevalence of hypertension. Meanwhile, a non-parametric test was used to assess differences in the distributions of gender and height between the two groups due to their abnormal distributions. Then, multivariable linear regression analysis was applied to detect the effect of intervention on the difference of hippocampal volumes and cognitive scores. In the linear regression model, we included age and gender in addition to the group as the independent variables. The results were reported as regression coefficient β, 95% confidence interval (CI), and associated *p*-value. All *p*-values were two-sided, and statistical significance was set at *p* = 0.05. In addition, the intraclass correlation coefficient (ICC; Shrout and Fleiss, 1979; Worker et al., 2018) was used to manually and semi-automatically compare hippocampal volume outputs at each time point.

## Results

### Participants

**FIGURE 1:**
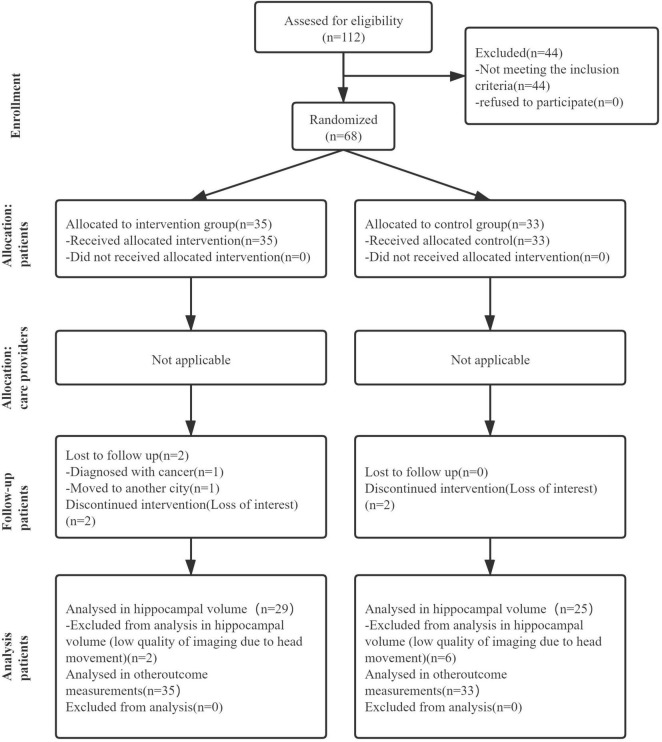
The flow diagram of the research, showing the experimental process of this article which includes recruitment process, grouping process, and subsequent data analysis.

Figure 1 shows the Consolidated Standards of Reporting Trials (CONSORT), highlighting the whole process from screening to the end of the study. We assessed 112 candidates who complained of loss of memory and other cognitive declines. Forty-four candidates were excluded according to the inclusion and exclusion criteria. Thus, 68 participants were included in this study and were randomly assigned to either the intervention group (*n* = 35) or the control group (*n* = 33). Descriptive statistics about randomized participants at baseline are summarized in Table 1.

**TABLE 1:** Patient characteristics at baseline.

Characteristics	Intervention Group (*n* = 35)	Control Group (*n* = 33)	Group-wise comparison *P*-value
Age (years), mean (SD)	71.51 (6.62)	69.82 (7.74)	0.334
Female, n (%)	18(51.42%)	23(70.00%)	0.055^a^
Height (cm), mean (SD)	157.17(8.50)	156.02(8.57)	0.215^a^
Weight (kg), mean (SD)	57.78(8.50)	57.47(10.61)	0.836
High Blood pressure^+^, n (%)	5(14.29%)	6(18.18%)	0.589
Hachinski Ischemia Score			0.084
0	13(37.14%)	7(21.21%)	
1	15 (42.86%)	13 (39.39%)	
2	2(5.71%)	8 (24.24%)	
3	4(11.43%)	5(15.15%)	
4	1(2.86%)	0(0.00%)	
Education years, mean (SD)	10.49(3.94)	9.49(4.14)	0.415

*SD, standard deviation.*

*^a^Nonparametric test was applied in non-normally distributed continuous data.*

*^+^High blood pressure is defined as systolic blood pressure >140 mmHg or diastolic blood pressure >90 mmHg.*

Sixty-two of the 68 participants completed the 3-month dance intervention and follow-up assessment. A total of 36 dance sessions were carried out, with a mean attendance rate of 88.6%. During the trial, two participants in the control group dropped out because of a loss of interest. In addition, four participants in the intervention group dropped out for the following reasons: one participant was diagnosed with cancer and advised for surgery, one participant moved to another city and could not attend dance sessions, and the other two participants lost interest in the study. Fortunately, no adverse events were reported during the whole duration of this study.

### Hippocampal Volume

Data of hippocampal volume we analyzed come from 29 participants in the intervention group and 25 participants in the control group at baseline and after intervention. Six participants refused to accept MRI scanning for personal affairs clashing. Eight participants were excluded from hippocampal volume calculation due to low-quality images caused by excessive head movement during MRI scanning. The right, left, and total hippocampal volumes are shown in Table 2. A linear regression model displayed the association between intervention and the difference of hippocampal volumes at baseline and after 3 months. The intervention group showed greater right hippocampal volume (β [95% CI]: 0.379 [0.117, 0.488], *p* = 0.002) and total hippocampal volume (β [95% CI]: 0.344 [0.082, 0.446], *p* = 0.005) compared to the control group. However, no significant association of age or gender was found in unilateral or global hippocampal volume.

**TABLE 2:** Linear regression model of hippocampal volumes and its potential confounders.

Variable	β (95% CI)	*P*
**Hippocampus volume (right)**		
Group	**0.379(0.117, 0.488)**	**0.002**
Age	−0.159(−0.022, 0.004)	0.170
Gender	−0.091(−0.263, 0.115)	0.437
**Hippocampus volume (left)**		
Group	−0.092(−0.143, 0.067)	0.473
Age	0.074(−0.005, 0.009)	0.557
Gender	0.014(−0.101, 0.113)	0.910
**Hippocampus volume (total)**		
Group	**0.344(0.082, 0.446)**	**0.005**
Age	−0.125(−0.019, 0.006)	0.288
Gender	−0.086(−0.254, 0.118)	0.468

*CI, confidence interval.*

*Multivariable linear regression models with right hippocampal volume, left hippocampal volume, and total hippocampal volume.*

*The model was adjusted by group, age, and gender.*

*Values in bold are statistically significant, i.e., p-values < 0.05.*

In addition, there was minimal shrinkage of the left hippocampus in the intervention group, although there was no inter-group difference (*p* = 0.46, 95% CI: −0.06, 0.13).

All calculated ICC scores were greater than 0.75 of hippocampal volume measured by the semi-automated and manual segmentation methods at each time point, and the results of the semi-automated and manual segmentation methods were significantly correlated. The reason we used manually measured hippocampal volume for analysis despite it being time-consuming and laborious is that it is recognized as the gold standard for measuring hippocampal volume (Frisoni et al., 2010; Dill et al., 2015).

### Cognition and Other Outcome Measurements

We used the linear regression model to examine the association between intervention and the change of cognitive scores, adjusting by age and gender (Table 3). There was a correlation between the intervention and episodic memory, as the intervention group showed high scores in WMS-RLM (β [95% CI]: 0.326 [1.005, 6.773], *p* = 0.009). Furthermore, an increase in age was associated with a decrease in MMSE score (β [95% CI]: −0.366 [−0.151, −0.034], *p* = 0.002). Other outcomes, including FAQ, SF-36, and GDS, are also summarized in Table 3. We established linear regression models using grouping, age, and gender, and found no associations of intervention with functional activities, quality of life, or depression. In addition, no association was found between age and other outcomes except MMSE.

**TABLE 3:** Linear regression of clinical assessments and its potential confounders.

Variable	β (95% CI)	P
**MMSE**		
Group	−0.021(−0.923, 0.768)	0.856
Age	−**0.366(**−**0.151,**−**0.034)**	**0.002**
Gender	−0.199(−1.596, 0.127)	0.094
**MoCA**		
Group	**0.280(0.159, 2.361)**	**0.026**
Age	−0.076(−0.100, 0.052)	0.533
Gender	−0.029(−1.253, 0.991)	0.816
**WMS-RLM**		
Group	**0.326(1.005, 6.773)**	**0.009**
Age	0.010(−0.191, 0.208)	0.934
Gender	0.004(−2.888, 2.991)	0.972
**SDMT**		
Group	0.038(−1.475, 1.991)	0.767
Age	0.034(−0.104, 0.136)	0.791
Gender	−0.057(−2.166, 1.366)	0.653
**TMTA**		
Group	−0.159(−18.733, 4.204)	0.210
Age	0.125(−0.391, 1.195)	0.315
Gender	−0.070(−14.962, 8.414)	0.578
**TMTB**		
Group	−**0.248(**−**62.506,**−**0.278)**	**0.048**
Age	−0.074(−2.806, 1.498)	0.546
Gender	−0.124(−47.726, 15.693)	0.317
**DST**		
Group	0.154(−1.728, 7.217)	0.225
Age	−0.060(−0.384, 0.234)	0.630
Gender	−0.096(−6.312, 2.805)	0.445
**GDS**		
Group	0.083(−2.136, 4.225)	0.514
Age	0.088(−0.142, 0.298)	0.483
Gender	−0.086(−4.344, 2.139)	0.499
**SF36**		
Group	0.137(−3.180, 10.711)	0.283
Age	0.001(−0.478, 0.483)	0.991
Gender	0.057(−5.480, 8.678)	0.653
**FAQ**		
Group	0.044(−0.979, 1.397)	0.726
Age	0.156(−0.030, 0.134)	0.210
Gender	−0.117(−1.781, 0.640)	0.350

*CI, confidence interval; MMSE, mini-mental state examination; MoCA, montreal cognitive assessment; WMS-RLM, Wechsler memory scale-revised logical memory; TMTA, trail making test part A; TMTB, trail making test part B; SDMT, symbol digit modalities test; DST, forward and backward digit span task; SF36, quality of life was measured by short-form health survey; FAQ, abilities of daily living were measured by functional activities questionnaire.*

*Multivariable linear regression models with clinical assessments.*

*The model was adjusted by group, age, and gender.*

*Values in bold are statistically significant, i.e., p-values < 0.05.*

## Discussion

### Aerobic Dance and Hippocampal Volume

This randomized clinical trial contributes new findings on the effects of aerobic dance on hippocampal volume and cognition in older adults with aMCI. The key finding was that aerobic dance increased right hippocampal volume and improved cognitive function in participants with aMCI.

Right hippocampal volume was increased by 11.2%, and total hippocampus volume was increased by 4.5%, which is corroborated by previous studies reporting volume increase in bilateral hippocampus among the elderly (ten Brinke et al., 2015; Teixeira et al., 2018). Another study of patients with multiple sclerosis revealed that hippocampal volume was preserved in the intervention condition of walking training, compared with hippocampal atrophy in the control condition (Sandroff et al., 2021). In our study, among patients with aMCI, we found a decrease in the volume of the left hippocampus by 2.61% in the intervention group and 5.88% in the control group.

There are two possible reasons for the increase in the right hippocampal volume. First, and most importantly, we used a different exercise protocol compared with other studies, which could possibly lead to more significant effects on the hippocampus. In our study, the aerobic dance consisted of physical movements that require abundant spatial stimulation, leading to an increased activation of the hippocampus, which appears to be involved in memory consolidation during locomotion (Burgess et al., 2002). The hippocampal and entorhinal networks are activated, and the place cells in the hippocampus fire in response to a unique, specific position in the environment (Foster, 2013). Therefore, dancing may provide a benefit by input stimulation following a simple exploration in motion of the surrounding environment. Second, the aerobic dance intervention we applied included a progress of repeated logical learning and review. A study on cognitively normal older adults pointed out that hippocampal volume was bilaterally related to landmark location learning and delayed memory (Sodoma et al., 2021). When we learn, the hippocampus, as an important part of the medial frontal lobe, is necessary for rapid consolidation and initial storage of episodic and semantic memory. The learning progress might increase the hippocampal volume, and the different performance of the bilateral hippocampus may be due to the different functions of the two parts. The right hippocampus is considered to be related to immediate and delayed recall (Yoo et al., 2019), visuospatial memory, as well as encoding spatial memory, whereas the left hippocampus is more involved in episodic verbal memory (Burgess et al., 2002; Ezzati et al., 2016; van Geest et al., 2018). This might mean that the aerobic dance we used requires a great deal of learning and memory processing of spatial information, which requires the involvement of the right hippocampus, leading to a significant increase in its volume.

### Age and Hippocampal Volume

Many studies on the hippocampal body and parahippocampal cortex have shown consistent age-related volume decline (Kennedy and Raz, 2009; Henson et al., 2016; Gorbach et al., 2017). However, we did not observe a similar significant change in hippocampal volume with age, which may be due to the insufficient age span or small sample size of our participants. At the same time, in the process of searching the literature, we found that there are relatively few studies on hippocampal volume, including the higher end of the age span, which must be remedied in our future research to explore the effect of age on hippocampal volume.

### Aerobic Dance and Cognition

A number of clinical trials have provided evidence that aerobic dance training can enhance cognition, including global cognition (Song et al., 2018), memory, and executive function in older adults with MCI (Nuzum et al., 2020). Similarly, our study demonstrated that 3 months of aerobic dance intervention could probably improve episodic memory in patients with aMCI, which is reflected in the higher scores of WLS-RLM. As our previous meta-analysis found, the aerobic dance could improve global cognitive function and memory performance of patients with aMCI (Zhu et al., 2020). This might be because the dance routine in our study involved a variety of movements in a specific order, so performing the routine required many cognitive efforts, such as initiation, orientation, and concentration, and particularly challenged episodic memory. Furthermore, as improvements in episodic memory and executive function were the key benefits to participants with aMCI in our study, aerobic dance might be a good intervention measure for patients with logical memory impairment in the future.

### Age and Cognition

A recent study on elderly people of Chinese descent in Singapore found that increasing age is a risk factor for cognitive impairment (Liu et al., 2022). We obtained similar results. As shown in Table 3, the MMSE score decreased with age, but no significant association with age was found for the other neuropsychological tests. For the safety of the completion process of aerobic dance, we did not collect data from very elderly people (the average ages of the intervention group and the control group were 71.51 ± 6.62 and 69.82 ± 7.74, respectively). However, this meant that the cognitive function of the participants had not declined significantly, which was not ideal for our study. Our future research needs to explore the changes of cognitive function in a more complete age stratification.

### Benefits of Aerobic Dance on Cognition and Its Possible Mechanism in the Elderly

Recently, different types of aerobic dance training have been used as interventions for elderly people in multiple studies. Many studies have confirmed that aerobic dance can improve cognitive function. Specially designed aerobic dances that combine physical exercise with cognitive tasks, similar to the one used in this study, merit further investigation in future studies (Murillo-Garcia et al., 2021). Several studies suggested that motor cognitive training has superior effects on cognition in older adults compared to single aerobic training or cognitive training alone (Rahe et al., 2015; Tait et al., 2017; Joubert and Chainay, 2018). The combination of aerobic exercise and cognitive effort in the dance program means that it can be classified as motor cognitive training, and it may improve cognition in the following ways. First, chronic aerobic exercise may improve cardiovascular fitness, which promotes long-term angiogenesis and cerebral circulation. This adaptation is related to increased delivery and upregulation of neurotrophins, supporting factors to the brain, particularly to the hippocampal neurogenic niche. Second, by combining aerobic exercise with cognitive training, motor cognitive training could provide long-term cognitive benefits and protection against age-related cognitive decline (Stimpson et al., 2018).

### Limitations

Several limitations have been recognized in this study. First, the participants in this study were highly educated people (the average education years of the intervention group and control group were 10.49 ± 3.94 and 9.49 ± 4.14, respectively), which could cause a selection bias from the main population with aMCI. Second, one of the methods of hippocampal volume calculation in our study was manual, which is not as convenient as a newly designed measuring software (Ahdidan et al., 2015; Frisoni et al., 2015) with an automatic segmentation algorithm for hippocampal volume calculation. Therefore, for future studies, we strongly recommend using professional software to measure hippocampal volume based on the consensus standard of hippocampus segmentation and measuring the volume by referring to the standard database.

## Conclusion

This study shows that 3 months of aerobic dance not only increases the volume of the hippocampus (right) but also improves cognitive function, especially episodic memory, in patients with aMCI. This means that aerobic dance has great potential for enhancing cognitive function by increasing hippocampus volume. However, further studies using functional MRI and positron emission tomography (PET) are needed to explore the mechanism of cognitive improvement by aerobic dance.

## Data Availability Statement

The original contributions presented in the study are included in the article/supplementary material, further inquiries can be directed to the corresponding authors.

## Ethics Statement

The Ethics Committee of the First Affiliated Hospital of Nanjing Medical University approved the study in January 2013(2012-SR-098). The patients/participants provided their written informed consent to participate in this study.

## Author Contributions

YZ: funding application, study management and coordination, and initial manuscript draft. ToW: providing research ideas and manuscript revision. LinZ: hippocampal volume calculation and manuscript revision. YG and CG: patient screening and inclusion and manuscript draft. MQ: MRI scanning protocol design and manuscript revision. HW: ethical application, assessments of participants, and manuscript draft. JM and LT: study design, statistical analysis, and revision of the manuscript. QiaZ and CS: intervention for the participants and manuscript draft. HD: MRI scanning and manuscript revision. NA: data analysis and manuscript draft. QiuZ: cognitive assessments, data analysis, and manuscript revision. LiZ and QinZ: data collection of baseline characteristics and follow-up, and manuscript revision. TiW and WW: study design, inclusion and exclusion criteria, patient diagnosis, and manuscript revision. All authors contributed to the article and approved the submitted version.

## Conflict of Interest

The authors declare that the research was conducted in the absence of any commercial or financial relationships that could be construed as a potential conflict of interest.

## Publisher’s Note

All claims expressed in this article are solely those of the authors and do not necessarily represent those of their affiliated organizations, or those of the publisher, the editors and the reviewers. Any product that may be evaluated in this article, or claim that may be made by its manufacturer, is not guaranteed or endorsed by the publisher.

## Footnotes

1
https://www.youtube.com/watch?v=WuIv1enhtL0
